# Synthesis, characterization, and magnetic properties of monodisperse CeO_2_ nanospheres prepared by PVP-assisted hydrothermal method

**DOI:** 10.1186/1556-276X-7-425

**Published:** 2012-07-31

**Authors:** Sumalin Phokha, Supree Pinitsoontorn, Prae Chirawatkul, Yingyot Poo-arporn, Santi Maensiri

**Affiliations:** 1 Department of Physics, Faculty of Science, Khon Kaen University, Khon Kaen, 40002, Thailand; 2 Synchrotron Light Research Institute (Public Organization), Suranaree University of Technology, Nakhon Ratchasima, 30000, Thailand; 3 School of Physics, Institute of Science, Suranaree University of Technology, Nakhon Ratchasima, 30000, Thailand

**Keywords:** CeO_2_, Nanospheres, Dilute magnetic oxide, Ferromagnetism, Oxygen vacancies, Valence states

## Abstract

Ferromagnetism was observed at room temperature in monodisperse CeO_2_ nanospheres synthesized by hydrothermal treatment of Ce(NO_3_)_3_·6H_2_O using polyvinylpyrrolidone as a surfactant. The structure and morphology of the products were characterized by X-ray diffraction (XRD), Raman spectroscopy, transmission electron microscopy, high-resolution transmission electron microscopy, and field-emission scanning electron microscopy (FE-SEM). The optical properties of the nanospheres were determined using UV and visible spectroscopy and photoluminescence (PL). The valence states of Ce ions were also determined using X-ray absorption near edge spectroscopy. The XRD results indicated that the synthesized samples had a cubic structure with a crystallite size in the range of approximately 9 to 19 nm. FE-SEM micrographs showed that the samples had a spherical morphology with a particle size in the range of approximately 100 to 250 nm. The samples also showed a strong UV absorption and room temperature PL. The emission might be due to charge transfer transitions from the 4*f* band to the valence band of the oxide. The magnetic properties of the samples were studied using a vibrating sample magnetometer. The samples exhibited room temperature ferromagnetism with a small magnetization of approximately 0.0026 to 0.016 emu/g at 10 kOe. Our results indicate that oxygen vacancies could be involved in the ferromagnetic exchange, and the possible mechanism of formation was discussed based on the experimental results.

## Background

Oxide-dilute magnetic semiconductors (O-DMSs) such as ZnO, TiO_2_, SnO_2_, and In_2_O_3_ doped with transition metal (TM) ions have recently attracted much attention due to their potential use in magneto-optoelectronic applications [1-3]. These O-DMSs are optically transparent and exhibit ferromagnetism (FM) at room temperature (RT) and even well above RT. Recently, TM-doped CeO_2_ have been also reported to exhibit ferromagnetism at and above room temperature [4-10]. Unlike other O-DMSs, CeO_2_ has a cubic structure with a lattice parameter *a* = 0.54113 nm [11] that will facilitate the integration of spintronic devices with advanced silicon microelectronic devices.

Early work on CeO_2_-based O-DMSs was focused on thin films [4-6] and only a few works have been carried out on powders, bulk, or nanocrystalline form [9-12]. Tiwari et al. [4] firstly discovered room temperature ferromagnetism (RT-FM) in Ce_1−*x*_Co_*x*_O_2−*δ*_ (*x* ≤ 0.05) films deposited on a LaAlO_3_ (001) substrate by pulsed laser deposition (PLD) technique. These films are transparent in a visible regime and exhibit a very high Curie temperature (*T*_C_) at approximately 740 to 875 K with large magnetic moments of 6.1 ± 0.2 to 8.2 ± 0.2 *μ*_*B*_/Co. Following the work by Tiwari et al., Song et al. [5] reported successful fabrication ofCe_1−*x*_Co_*x*_O_2−*δ*_ (*x* = 0.03) thin films with (111) preferential orientation deposited on a Si (111) substrate by a PLD technique. Their deposited films show RT-FM with large magnetic moment of 5.8 *μ*_*B*_/Co and coercivity of 560 Oe. The authors also showed that the films could be deposited on glass but with smaller magnetic moment and coercivity. These results suggested that the FM in Co-doped CeO_2_ depend not only on the doping concentration of transition element, but also on the microstructure of film, including its crystallization, defects, vacancies, etc. Vodungbo et al. [6] also reported FM in Co-doped CeO_2_ thin films grown by PLD on SrTiO_3_ and Si substrate. The films were ferromagnetic with a *T*_C_ above 400 K. These authors found that the amount of structural defects had a little effect on FM, but the presence of oxygen during the growth or annealing reduced drastically the FM, suggesting that oxygen vacancies played an important role in the magnetic coupling between Co ions, while Wen et al. [9] reported the ferromagnetism observed in pure and Co-doped CeO_2_ powders. The RT-FM in pure CeO_2_ originated from oxygen vacancies while a slight Co doping in CeO_2_ caused a nearly two-order enhancement of saturation magnetization (*M*_s_) to 0.47 emu/g as compared with the pure sample. The authors suggested that the large RT-FM observed in Co-doped CeO_2_ powder originated from a combination effect of oxygen vacancies and Co doping. Similarly, Ou et al. [10] reported RT-FM for Ce_1−*x*_Co_*x*_O_2_ (0 < *x* < 0.10) nanorods prepared by electrodeposition route. The nanorods were ferromagnetic with a high *T*_C_ of about 870 K and the largest *M*_s_ of 0.015 emu/g. They suggested that the RT-FM observed in Co-doped CeO_2_ nanorods was adjusted by the structural defects including oxygen vacancies. The same behavior was found in nanoparticles of Fe-doped CeO_2_[11] with an *M*_s_ value of 0.0062 emu/g in 3 at % Fe prepared by a sol–gel method and Fe-doped CeO_2_[12] with an *M*_s_ value of 0.10 emu/g in 1 at % Fe prepared by the proteic sol–gel process. The authors suggested that the RT-FM originated from an exchange of *F*-center, which involved a combination of oxygen vacancies and TM doping.

Surprisingly, the researchers report RT-FM of undoping in different oxides, such as thin films of HfO_2_[13], TiO_2_ and In_2_O_3_[14], and nanoparticles of CeO_2_, Al_2_O_3_, ZnO, In_2_O_3_, and SnO_2_[15], while the corresponding bulk samples are diamagnetic. Most recently, there are some studies reporting ferromagnetism observed in pure CeO_2_ on powders, nanocrystalline, or cubes [16-18]. Liu et al. [16] studied the size-dependent ferromagnetism in CeO_2_ powders synthesized by precipitation route. They found that ferromagnetism was observed only in sub-20-nm powders with an *M*_s_ value of 0.08 emu/g. Similarly, Chen et al. [17] reported RT-FM in CeO_2_ nanoparticles prepared by thermal decomposition method with an *M*_s_ value of 0.12 emu/g. The authors showed that its crystallite size in nanometers would be ferromagnetic because of the large value of the surface-to-volume ratio, leading to the exchange interactions between electron spin moments that resulted from oxygen vacancies at the surface [4]. Recently, Ge et al. [18] observed ferromagnetism in CeO_2_ nanocubes with an *M*_s_ value of 0.0057 emu/g (an average size of 5.3 nm) prepared by a chemical method. They suggest that oxygen vacancy is essential for the formation of FM in CeO_2_ nanocubes.

However, magnetic properties of monodisperse nanospheres of pure CeO_2_ have not yet been reported. In this work, we report the ferromagnetism observed in monodisperse CeO_2_ nanospheres with a particle size of approximately 200 nm synthesized by hydrothermal treatment of Ce(NO_3_)_3_·6H_2_O using polyvinylpyrrolidone (PVP) as a surfactant. The technique of preparation and the effect of the type of cerium source on the crystallinity and morphology were investigated. The prepared samples were characterized by X-ray diffraction (XRD), Raman spectroscopy, field-emission scanning electron microscopy (FE-SEM), transmission electron microscopy (TEM), high-resolution transmission electron microscopy (HRTEM), UV and visible spectroscopy (UV–vis), and photoluminescence (PL). The valence states of Ce ions were also investigated by using X-ray absorption near edge spectroscopy (XANES), and the magnetic properties of the samples were determined using a vibrating sample magnetometer (VSM). The origin of RT-FM in this pure CeO_2_ is also discussed.

## Methods

In this study, cerium (III) nitrate hexahydrate, Ce(NO_3_)_3_·6H_2_O (99.99% purity; Kanto Corporation, Portland, OR, USA); cerium (III) acetate hydrate, Ce(CH_3_CO_2_)_3_·*x*H_2_O (99.9% purity; Sigma-Aldrich Corporation, St. Louis, MO, USA); cerium (III) chloride heptahydrate, CeCl_3_·7H_2_O (99.9% purity; Sigma-Aldrich Corporation); cerium (III) sulfate octahydrate, Ce_2_(SO_4_)_3_.8H_2_O (99.999% purity; Sigma-Aldrich Corporation); and PVP (Sigma-Aldrich Corporation) were used as starting materials. In a typical procedure, one gram of PVP was mixed with 40 mL of deionized water under vigorous magnetic stirring at room temperature (27°C) until a homogeneous solution was obtained. Subsequently, 3 mmol of cerium source was slowly added to the PVP solution under vigorous stirring at room temperature for 2 h, in order to obtain a well-dissolved solution. Throughout the whole process described, no pH adjustment was made. The homogeneous solution was transferred into a Teflon-lined stainless steel autoclave of 50-mL capacity and prepared at 160°C and 200°C for 12 h and 160°C and 200°C for 24 h. After the autoclave was cooled naturally to room temperature, the precipitate was collected and washed several times with distilled water. The final product was then dried in a vacuum at 80°C overnight. In addition, the as-prepared samples were also annealed in argon atmosphere at 400°C for 2 h to study the effect of oxygen vacancies on magnetic properties of the annealed samples.

The prepared samples were characterized using XRD, Raman spectroscopy, FE-SEM, TEM, HRTEM, UV–vis, PL, XANES, and VSM. A Philips X-ray diffractometer (Philips Tecnai, Amsterdam, The Netherlands) with CuKα radiation (*λ* = 0.15406 nm) was used to study the phases of the pure CeO_2_ samples. The Raman spectra were recorded at room temperature using a triple spectrometer (Jobin Yvon/Atago-Bussan T-64000, HORIBA Jobin Yvon S.A.S., Chilly-Mazarin, France). The morphology of the sample was obtained from TEM (JEM 2010 200 kV, JEOL Ltd., Akishima, Tokyo, Japan). FE-SEM was performed using a JEOL JSM-6335 F (JEOL Ltd.). The optical absorption spectrum was measured in the range of 200 to 800 nm using a UV-3101PC UV–vis-NIR scanning spectrometer (Shimadzu Corporation, Nakagyo-ku, Kyoto, Japan). PL was carried out on a luminescence spectrometer (PerkinElmer LS-55B, PerkinElmer Instrument, Waltham, MA, USA), using a Xenon lamp as the excitation source at room temperature. The Ce L_3_ XANES spectrum was studied using XANES in transmission mode at the BL4 Station at Siam Photon Laboratory (Synchrotron Light Research Institute (Public Organization), SLRI) in Nakhon Ratchasima, Thailand. The magnetic measurements were performed at room temperature using a vibrating sample magnetometer (VSM 7403, Lakeshore, Westerville, OH. USA).

## Results and discussion

### XRD analysis

**Figure 1:**
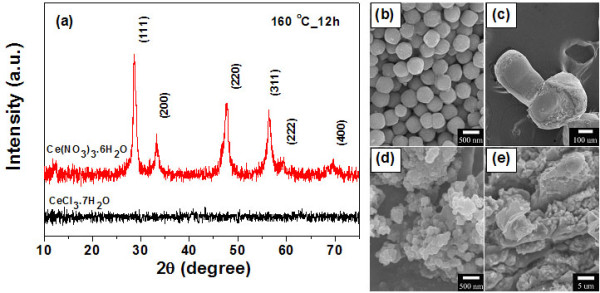
**XRD pattern and FE-SEM images of the cerium source.** (**a**) XRD patterns of cerium source as the starting materials prepared at 160°C for 12 h Ce(NO_3_)_3_·6H_2_O and CeCl_3_·7H_2_O. FE-SEM images of cerium source as the starting materials prepared at 160°C for 12 h (**b**) Ce(NO_3_)_3_·6H_2_O, (**c**) CeCl_3_·7H_2_O, (**d**) Ce(CH_3_CO_2_)_3_·*x*H_2_O, and (**e**) Ce_2_(SO_4_)_3_·8H_2_O.

The XRD patterns of the samples prepared by hydrothermal reaction at 160°C for 12 h are shown in Figure 1a. The sample obtained with CeCl_3_·7H_2_O as the starting agent shows no XRD peaks, indicating that it is amorphous, whereas the sample from Ce(NO_3_)_3_·6H_2_O exhibits XRD peaks that correspond to the (111), (200), (220), (311), (222), and (400) planes, which are consistent with the face-centered cubic fluorite structure of CeO_2_ in the standard data from the Joint Committee on Powder Diffraction Standards (JCPDS) 34–0394, indicating that pure CeO_2_ was successfully synthesized via these procedures.

In this study, we found that the type of cerium source has a great effect on the morphology of the final product. The cerium source from Ce(NO_3_)_3_·6H_2_O shows that the sample consisted of sphere-like particles with diameters of 100 to 250 nm (Figure 1b), whereas other cerium sources such as CeCl_3_·7H_2_O, Ce(CH_3_CO_2_)_3_·*x*H_2_O, and Ce_2_(SO_4_)_3_·8H_2_O resulted in irregular shapes and agglomerated particles as shown in Figure 1c,d,e, respectively. Therefore, it is clearly seen that cerium source from nitrate is most favorable for the formation of uniformly sized CeO_2_ nanospheres. It is possible that the absorption of PVP molecules on various crystallographic planes of cerium source played a major role in determining the product morphology, due to the fact that the supersaturation degree has a significant influence on the crystal nucleation rate and crystal growth rate [19]. However, the real reason for the morphology variation of the cerium source and surfactants has yet to be fully understood.

**Figure 2:**
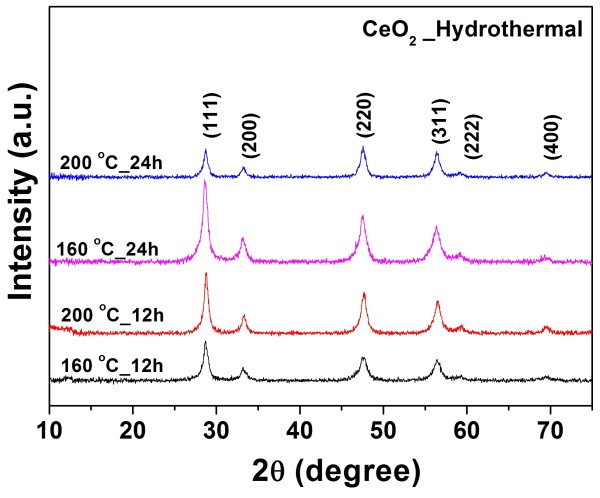
**XRD patterns of obtained CeO**_**2**_**nanospheres.** Ce(NO_3_)_3_·6H_2_O was used as starting materials prepared at 160°C and 200°C for 12 and 24 h.

**Figure 3:**
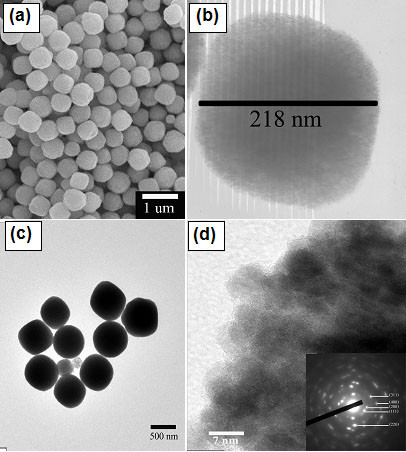
**FE-SEM and TEM bright field images of CeO**_**2**_**nanospheres.** (**a**) FE-SEM images of CeO_2_ nanospheres prepared at 200°C for 24 h; (**b**) TEM bright field images of CeO_2_ nanospheres prepared at 160°C for 12 h; (**c**) and (**d**) are TEM bright field images with corresponding SAED patterns (insets) of CeO_2_ nanospheres prepared at 200X°C for 12 h.

Figure 2 shows the XRD patterns of the pure CeO_2_ from cerium nitrate at various hydrothermal treatment durations and temperatures. All the samples exhibited six typical peaks corresponding to the (111), (200), (220), (311), (222), and (400) planes, which are consistent with the face-centered cubic fluorite structure of CeO_2_ in the standard data from JCPDS 34–0394, and this is in agreement with the selected area electron diffraction (SAED) patterns shown in Figure 3d. The values of the lattice constant calculated from the XRD spectra are shown in Table 1. The average crystallite size of all the samples was calculated from X-ray line broadening of the peaks at the (111), (200), (220), and (311) planes using Scherrer’s equation, (as listed in Table 1). We observed that the lattice parameter decreases with increasing crystallite size. This decrease is possibly due to the introduction of Ce^3+^ ions into the crystal lattice. Ce^3+^ ions have a higher ionic radius (1.034 Å) compared with the Ce^4+^ ions (0.92 Å) and introduce oxygen vacancies. Therefore, the concentration of Ce^3+^ ions increases, and there is also an increase in the number of oxygen vacancies. It is observed that the pure CeO_2_ nanoparticles experience considerable lattice distortion, which is in good agreement with earlier reports on CeO_2_ nanoparticles [9,20], which indicated that this causes a change in the Ce-O bond length (lattice distortion) and the overall lattice parameter.

**Table 1:** **Summary of crystallite sizes, lattice constant, bandgap, and magnetization of pure CeO**_**2**_**nanospheres**

**Sample**	**Crystallite size from XRD (nm)**	**Lattice constant*a*(nm)**	***E***_***g***_**(eV)**	**Crystallite size from Raman spectroscopy (nm)**	***M***_**s**_**at 10 kOe (emu/g)**	
					**Before Ar annealing**	**After Ar annealing**
CeO_2_ at 160 °C for 12 h	9.43 ± 0.41	0.5430 ± 0.0021	3.00	7.39	-	-
CeO_2_ at 200 °C for 12 h	19.6 ± 0.53	0.5420 ± 0.0003	3.04	8.21	0.0026	0.011
CeO_2_ at 160 °C for 24 h	12.2 ± 0.13	0.5430 ± 0.0003	3.06	9.23	0.0053	0.0026
CeO_2_ at 200 °C for 24 h	15.6 ± 0.20	0.5428 ± 0.0006	3.10	12.28	0.016	0.015

*E*_*g*_, bandgap energy; *M*_s_, saturation magnetization. Data of magnetization of pure CeO_2_ nanospheres is before and after Ar annealing.

### Raman analysis

**Figure 4:**
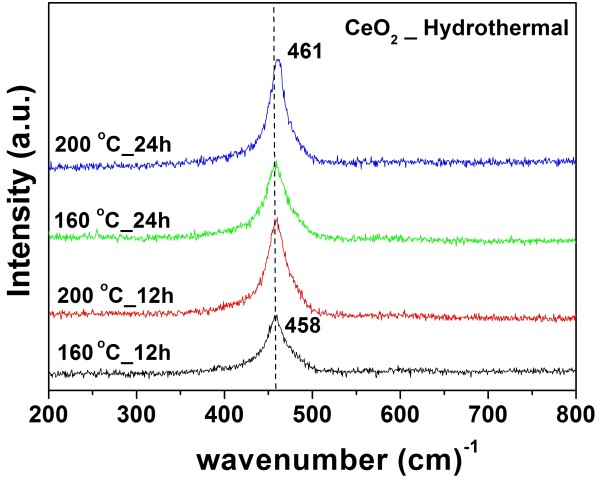
**Raman spectra of obtained CeO**_**2**_**nanospheres.** Ce(NO_3_)_3_·6H_2_O was used as starting materials prepared at 160°C and 200°C for 12 and 24 h.

The formation of a cubic structure in the CeO_2_ nanospheres was further supported by the Raman spectra. Figure 4 shows typical spectra of CeO_2_. The Raman active modes are shifted from 458 to 461 cm^−1^ for the CeO_2_ samples heated at 160°C to 200°C. These Raman active modes are attributed to a symmetrical stretching mode of the Ce-80 vibrational unit, and therefore, they are very sensitive to any disorder in the oxygen sublattice that resulted from thermal, doping, or grain size [21-24]. The effect of the microstructure of CeO_2_ on the shape of the Raman spectra was observed by the broadening of the line and by increases in its asymmetry, which are attributed to the reduction of the phonon lifetime in the nanocrystalline regime [25, 26]. The particle size of the CeO_2_ sample can be also estimated from the Raman line broadening using the following Equation 1 [23, 24, 27]:

(1) $$\Gamma ({\text{cm}}^{-1})=10+\frac{124.7}{D_{R}}\text{,}$$

where $\Gamma ({\text{cm}}^{-1})$ is the full width at half maximum of the Raman active mode peak and *D*_*R*_ is the particle size of a CeO_2_ sample. This relation can be used to determine the crystal size of the CeO_2_ samples as shown in Table 1. This calculated crystal size is near that obtained from X-ray line broadening.

### FE-SEM, TEM, and HRTEM analyses

**Figure 5:**
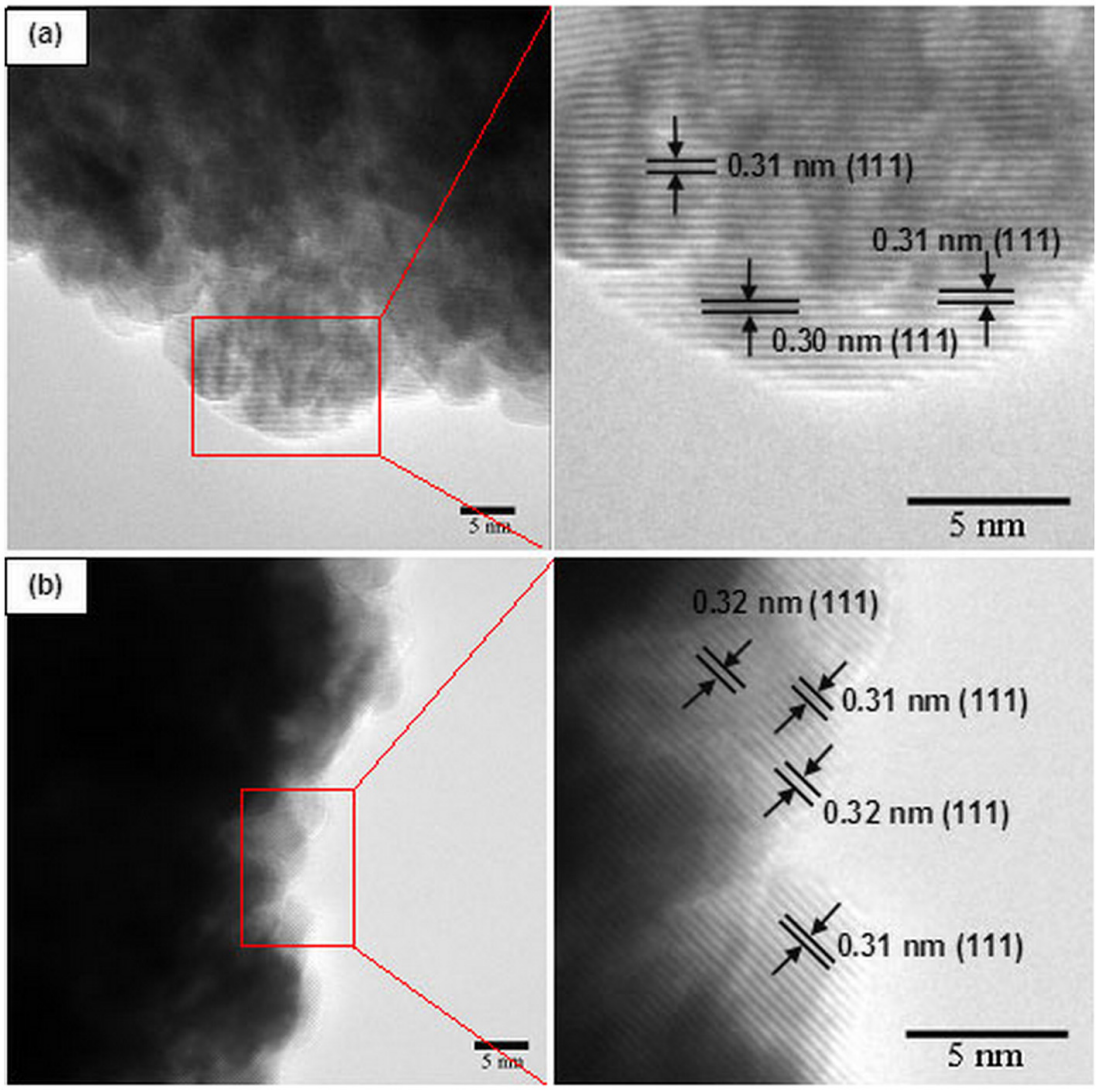
**HRTEM images of CeO**_**2**_**nanospheres prepared at 200°C for 24 h.** (**a**) As-prepared CeO_2_ nanospheres and (**b**) CeO_2_ nanospheres after annealing in Ar at 400°C for 2 h.

The morphology and structure of CeO_2_ nanospheres were investigated by FE-SEM and TEM as shown in Figure 3. TEM bright field images show that the samples contain monodisperse nanospheres with a narrow size distribution. The high-magnification TEM image of a single particle (Figure 3b) indicates that the sphere has a diameter of about 218 nm. This result is similar to the work reported by Zhou et al. [28], in which spherical CeO_2_ crystallites assembled by nanoparticles were synthesized by hydrothermal treatment because small nanoparticles of CeO_2_ aggregated and gradually evolved into a spherical assembly, achieving a low surface energy. The corresponding SAED patterns (inset in Figure 3d of the products show spotty ring patterns indicative of a face-centered cubic structure of CeO_2_ (JCPDS 34–0394), which is in agreement with the XRD results. The HRTEM images of the CeO_2_ sample prepared at 200°C for 12 h and the CeO_2_ sample prepared at 200°C for 12 h followed by annealing in Ar at 400°C for 2 h, are shown in Figure 5a,b, respectively. The *d* spacings of the lattice fringes of approximately 0.30 and 0.31 nm for the CeO_2_ sample prepared at 200°C for 12 h (Figure 5a) calculated from the HRTEM images correspond to the (111) plane of CeO_2_, whereas the *d* spacings of approximately 0.31 and 0.32 nm (Figure 5b) for the CeO_2_ sample prepared at 200°C for 12 h followed by annealing in Ar at 400°C for 2 h match with (111) plane of CeO_2_. This is in good agreement with the standard data (JCPDS 34–0394).

### Optical properties

**Figure 6:**
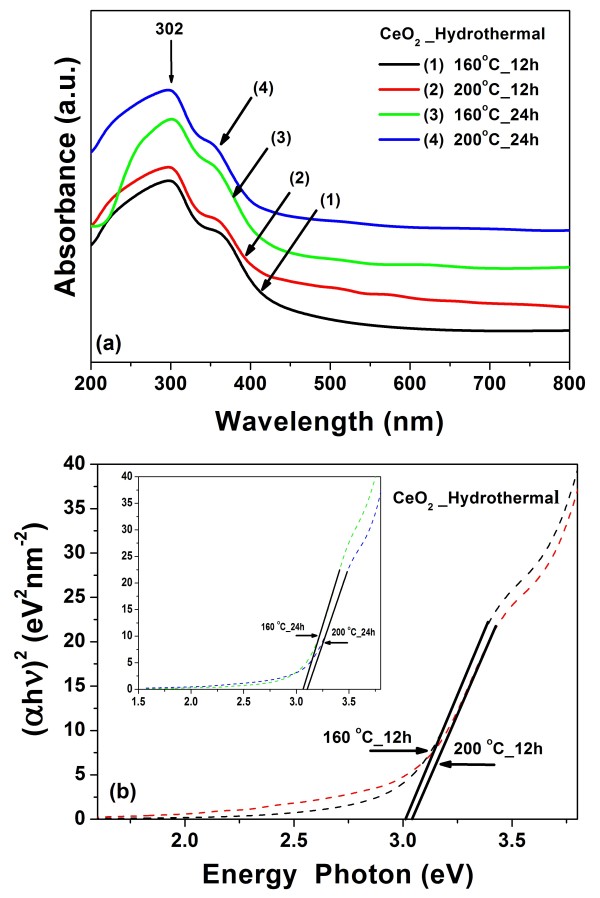
**Room temperature optical absorbance spectra of CeO**_**2**_**nanospheres and plot of (α*h*v)**^**2**^**.** (**a**) Room temperature optical absorbance spectra of CeO_2_ nanospheres using Ce(NO_3_)_3_·6H_2_O as starting materials. (**b**) Plot of (*α*hv)^2^ as a function of photon energy for the CeO_2_ nanospheres.

The UV–vis absorption spectra of the pure CeO_2_ nanospheres are shown in Figure 6a. All the samples show a strong absorption below 400 nm (3.10 eV) with a well-defined absorbance peak at approximately 302 nm (4.10 eV). The direct bandgap energy (*E*_g_) is determined by fitting the absorption data to the direct transition as shown in Equation 2:

(2) $$\alpha \text{hν}=A{\left(\text{hν}-E_{\text{g}}\right)}^{1/2}$$

where *α* is the optical absorption coefficient, hν is the photon energy, *E*_g_ is the direct bandgap, and *A* is a constant [29]. The extrapolation of the linear portions of the curves towards absorption equal to zero (y = 0) gives *E*_g_ for direct transitions (Figure 6b). The estimated direct bandgaps of all the samples are shown in Table 1. The bandgap of CeO_2_ reported in this work is lower than that reported in the literature. Chen and Chang [30] reported direct bandgap values ranging from 3.56 to 3.71 eV for CeO_2_ nanoparticles synthesized by precipitation method. Maensiri et al. [21] reported direct bandgap values ranging from 3.57 to 3.61 eV for CeO_2_ nanoparticles synthesized by the sol–gel method using egg white. Similarly, Masui et al. reported direct bandgap values of 4.1 and 2.6 nm for CeO_2_ nanoparticles prepared using reverse micelles to be 3.38 and 3.44 eV [31], respectively, due to quantum confinement effect [32]. This phenomenon has been well explained for particle sizes down to less than a few nanometers, but for our results, the bandgaps increased with increasing crystal size, which exhibit blueshifts in the UV absorption spectra inferred from the bandgap calculated for pure CeO_2_ nanospheres. This blueshift has been reported to be an electrostatic potential effect due to a cerium valence change when the particle size is larger than a few nanometers (e.g., ≥8 nm). The Ce^4+^ ions coexist with Ce^3+^ ions, and these ions can be attributed to oxygen vacancies at the surface [33]. Therefore, in our work, the bandgaps increased with increasing crystal size. The crystallite size is in the range of 9 to 19 nm as indicated by the existence of the blueshift for our CeO_2_ nanospheres.

### PL analysis

**Figure 7:**
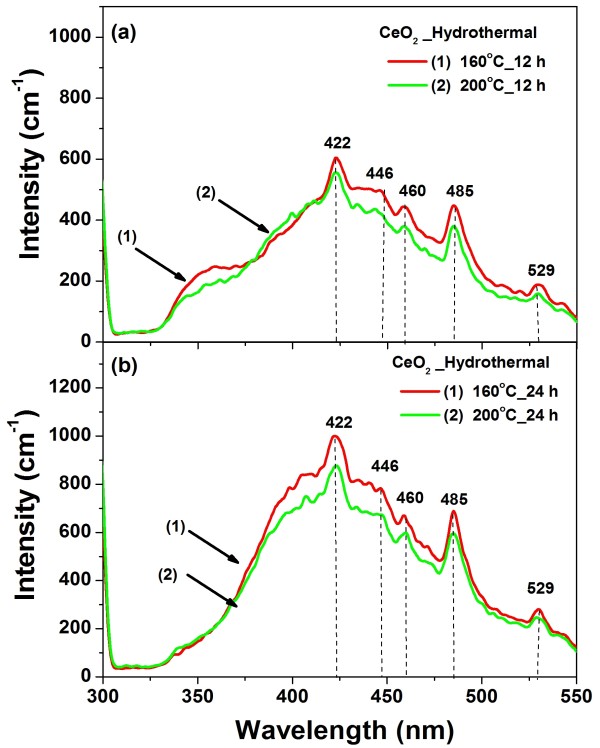
**Room temperature photoluminescence spectrum of CeO**_**2**_**nanospheres using Ce(NO**_**3**_**)**_**3**_**·6H**_**2**_**O as starting materials.** The sample was dispersed in methanol and the excitation wavelength used in PL measurement was 290 nm.

Figure 7 shows the room temperature PL spectra obtained using a Xenon laser of 290 nm as the excitation source of the CeO_2_ nanospheres. The spectra of all the samples are almost identical and mainly consist of five emission bands: a strong blue emission band at 422 nm (2.93 eV), a weak blue band at 446 nm (2.78 eV), a blue band at 460 nm (2.69 eV), a strong blue-green band at 485 nm (2.55 eV), and a green band at 529 nm (2.35 eV). Our results are consistent with that reported for CeO_2_ in the literature. The strong emission peak at 410 nm [34] and 422 nm [35] observed for CeO_2_ nanoparticles as-prepared at *λ*_ex_ = 290 nm. Maensiri et al. [21] reported a blue band at approximately 443 nm, along with a green band at 529 nm for 400°C to 500°C calcined sample, and a strong UV emission band at 392 nm for 600°C calcined sample. The dependence of the PL blueshift peak on CeO_2_ particle concentration has also been observed by Sathyamurthy et al. [34] for CeO_2_ nanoparticles synthesized by a reverse micelle route. This phenomenon has been explained by charge transitions from the 4*f* band to the valence band of the CeO_2_ in both nanoparticles and thin films [36]. In addition, it is well known that the emission energy from the 4*f* band to the valence band energy gap of CeO_2_ is about 3.0 to 3.38 eV, as determined from the calculation of the electronic structure of CeO_2_[35,37]. Therefore, the emission in our CeO_2_ samples could be assumed to be the transition from the Ce 4*f* band to the O 2*p* band (valence band) in CeO_2_. The broad PL band ranging from 300 to 550 nm of all the samples could be the result of defects, including oxygen vacancies in the crystal with electronic energy levels below the 4*f* band [38]. This is confirmed by the enhanced absorption tail below 3 eV in nonstoichiometric CeO_2_ that was previously observed and attributed to the presence of oxygen vacancies [39].

### XANES analysis

**Figure 8:**
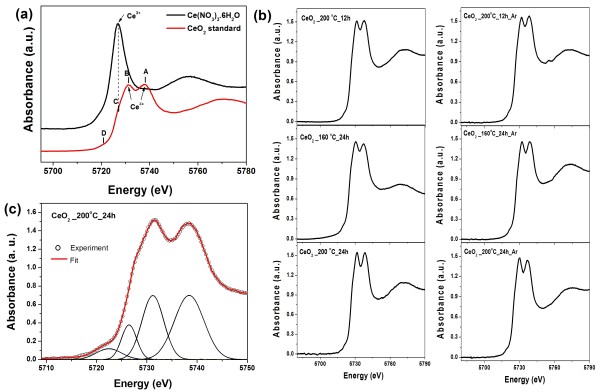
**XANES spectra for Ce(NO**_**3**_**)**_**3**_**·6H**_**2**_**O and CeO**_**2**_**standard and pure CeO**_**2**_**and Gaussian fitting of XANES spectra.** (**a**) XANES spectra at the Ce L_3_ absorption edge for Ce(NO_3_)_3_·6H_2_O and CeO_2_ standard showing features of A, B, C, and D. (**b**) XANES spectra of pure CeO_2_ at various hydrothermal treatment durations and temperature before and after annealing in Ar. (**c**) A Gaussian fitting of XANES spectra of pure CeO_2_ prepared at 20°C for 24 h.

The valence state of Ce in pure CeO_2_ nanospheres was determined by XANES spectra measured at Ce L_3_ edge. Figure 8a shows the edge energies of the Ce(NO_3_)_3_·6H_2_O standard and CeO_2_ standard. The standard Ce(NO_3_)_3_·6H_2_O has a single peak illustrated by one intense white line at approximately 5,726.8 eV, which can be associated with the final state of 2*p*4*f*^1^5*d*e_g_L, where L denotes an oxygen ligand 2*p* hole, corresponding to the Ce^3+^ valence state [40]. In the standard CeO_2_, there are four peaks comprising high energy peak A, main peak B, low energy peak C, and pre-edge peak D, which have been reported and assigned previously [41-43]. Peaks A and B are shifted to higher energies at approximately 5,737.9 and 5,731.3 eV, respectively, and were assigned as being due to a mixture of the multi-electron with the final state of 2*p*4*f*^0^5*d* and 2*p*4*f*^1^5*d*t_g_L, respectively, which characterizes the Ce in the Ce^4+^ valence state [40]. Peak C, at approximately 5,726.8 eV, is observed at the same energy as the white line of a typical Ce(NO_3_)_3_·6H_2_O standard, corresponding to the Ce^3+^ valence state. Peak D is assigned to the final states of 2*p*5*d* with a delocalized *d* character at the bottom of the conduction band due to the cubic crystal-field splitting of Ce 5*d* states [44].

Figure 8b shows the XANES spectra of pure CeO_2_ for various hydrothermal treatment durations and temperatures before and after annealing in Ar. The quantitative analysis of the valence state of Ce in each of the three states of the CeO_2_ nanospheres was performed using multi-peak Gaussian fitting obtained from the XANES spectra, as shown in Figure 8c. The analysis shows that Ce in the samples is in a mixed valence state of Ce^3+^ and Ce^4+^. From these results, we can obtain the valence state of Ce according to the fitting parameters of the peak positions and areas listed in Table 2. It is observed that the percentage of Ce^3+^ ranges from 7% to 13.7% in the pure CeO_2_ samples. These results provide confirmation of the formation of oxygen vacancies on the surface of the CeO_2_ samples. It is possible that the concentration and distribution of oxygen vacancies play an important role in the magnetism of our CeO_2_ nanospheres. The highest percentage of Ce^3+^ is 13.7% for CeO_2_ prepared at 200°C for 24 h, with the increase in the number of oxygen vacancies, leading to the highest *M*_s_ values. The concentration of Ce^3+^ in our pure CeO_2_ sample is higher than the values reported in the literature for CeO_2_. Zhang et al. [41] reported the average Ce^3+^ concentration of 10- and 6-nm CeO_2_ nanoparticles prepared by mixing cerium nitrate and hexamethylenetetramine in aqueous solution at room temperature to be 1% and 6.5%, respectively. Chen et al. [17] reported that the concentration of Ce^3+^ was higher than 21% for CeO_2_ nanoparticles synthesized by the thermal decomposition method, which is higher than the value for pure CeO_2_ nanospheres reported in this study.

**Table 2:** **Gaussian fitting for percentage of Ce**^**3+**^**of pure CeO**_**2**_**nanospheres before and after Ar annealing**

**Sample**	**Peak position (eV)**		**Peak area (eV)**		**Percentage of Ce**^**3+**^**(%)**	
	**Before Ar annealing**	**After Ar annealing**	**Before Ar annealing**	**After Ar annealing**	**Before Ar annealing**	**After Ar annealing**
CeO_2_ at 200 °C for 12 h	5737.881	5738.471	5.128	5.672	7.8	12.4
	5730.733	5731.222	3.791	3.603		
	5724.617	5725.931	0.761	1.308		
CeO_2_ at 160 °C for 24 h	5737.245	5738.888	6.734	4.703	9.8	10.4
	5729.911	5731.695	3.549	3.427		
	5726.128	5726.769	1.013	0.949		
CeO_2_ at 200 °C for 24 h	5738.440	5736.924	5.154	5.713	13.7	13.3
	5731.205	5729.691	3.799	3.741		
	5726.465	5724.987	1.427	1.445		

### Magnetic properties

**Figure 9:**
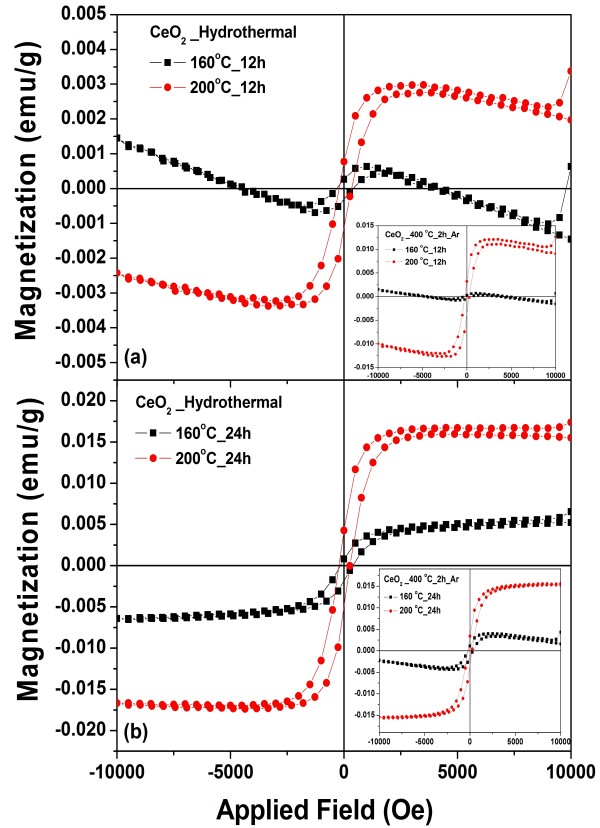
**Magnetic properties of monodisperse CeO**_**2**_**nanospheres.** Prepared at (**a**) 160°C and 200°C for 12 h and (**b**) 160°C and 200°C for 24 h. Inset shows CeO_2_ samples after annealing in argon atmosphere at 400°C for 2 h.

Figure 9a,b shows the field dependence of the specific magnetization (*M-H* curve) of pure CeO_2_ samples prepared at 160°C and 200°C for 12 h and prepared at 160°C and 200°C for 24 h, respectively, obtained from room temperature VSM measurements. The sample of CeO_2_ prepared at 160°C for 12 h exhibits mixed behaviors of ferromagnetism and diamagnetism having hysteresis loops at low field. The samples that were prepared at 200°C for 12 h showed weak RT-FM with magnetization (*M*) of approximately 0.0026 emu/g, and the samples that were prepared at 160°C and 200°C for 24 h showed weak RT-FM with saturation magnetization (*M*_s_) of approximately 0.0053 and 0.016 emu/g, respectively (as listed in Table 1). These values are higher than the *M*_s_ values reported in the literature for pure CeO_2_ nanospheres [15,17]. Sundaresan et al. [15] reported an RT-FM with an *M*_s_ value of approximately 0.0019 emu/g for CeO_2_ nanoparticles with an average size of approximately 15 nm. Ge et al. [18] reported weak ferromagnetic behavior at an ambient temperature with an *M*_s_ value of approximately 0.0007 emu/g for pure CeO_2_ nanoparticles with an average size of approximately 100 nm obtained commercially from Sigma-Aldrich Corporation (purity of 99.9%). However, magnetic behavior (*M*_s_ of approximately 0.0057 emu/g) was also observed in monodisperse CeO_2_ nanocubes with an average size of approximately 5.3 nm prepared by a chemical method. The results obtained here (experimental and theoretical) provide evidence that pure CeO_2_ samples can indeed have a magnetic moment due to oxygen vacancies. This direct ferromagnetic coupling is called *F*-center exchange (FCE) [45], as cerium can have both variable valence states (Ce^4+^/Ce^3+^) and oxygen vacancies on the surface of the CeO_2_ nanoparticles. It is possible that oxygen vacancies can create magnetic moments on neighboring Ce ions [46]. In this work, the effect of Ar annealing at 400°C for 2 h on the magnetic properties was also performed to confirm the effect of oxygen vacancies on magnetic properties of the annealed samples. However, this effect was clearly observed only on the CeO_2_ sample prepared at 200°C for 12 h and followed by annealing in argon atmosphere at 400°C for 2 h, as its magnetization increased from 0.0025 emu/g to 0.010 emu/g as (see inset of Figure 9b). It is noted that for the samples prepared at 160°C and 200°C for 24 h, annealing did not affect much their magnetic behavior due to the short time of annealing.

To explain the origin of the ferromagnetic contribution in the CeO_2_ nanostructures, the following arguments are proposed. The annealing of samples in an Ar atmosphere at 400°C for 2 h could possibly increase the number of oxygen vacancies and Ce^3+^ ions in the samples. The high concentration of Ce^3+^ (approximately 13.3% Ce^3+^ for the sample prepared at 200 °C for 24 h) suggests that defects could be present in the majority of the samples, which activate more coupling between the Ce ions, leading to an increase in *M*_s_. Wen et al. [9] reported the variation of RT-FM in oxygen and H_2_ (10%)/Ar (90%) annealed samples of 1% Co-doped CeO_2_ powder. They found that the sample showed little hysteresis loop after O_2_ annealing and that the FM signal decreased significantly, while the H_2_ (10%)/Ar (90%) annealed sample showed an enhanced FM with *M*_s_ of about 0.4 emu/g. However, further work is needed to achieve a thorough understanding, and this will be of great interest to researchers in the field of dilute magnetic oxides.

## Conclusions

In summary, spheres of pure CeO_2_ with Ce(NO_3_)_3_·6H_2_O using PVP as a surfactant have been successfully synthesized by hydrothermal method, and their structures, valence state, and magnetic properties were investigated. The XRD and Raman spectroscopy results suggested the formation of CeO_2_ cubic fluorite structures in the CeO_2_ samples, which was in agreement with the SAED patterns. It is observed that there is a decrease in the lattice parameters with increasing crystallite size, possibly due to the formation of structure defects/oxygen vacancies in the CeO_2_ lattice. The bandgaps of our CeO_2_ nanospheres increased with increasing crystal size indicated by the existence of a blueshift due to a cerium valence change, and this can be attributed to oxygen vacancies at the surface. The surface defects in the CeO_2_ nanospheres play an important role in the PL properties of our sample. The XANES results reveal that a fraction of the Ce ions are in the 3+ state, and these cause the samples to show weak RT-FM with an *M*_s_ value of 0.0026 to 0.016 emu/g. A ferromagnetic exchange mechanism in the pure CeO_2_ samples is discussed by FCE, and the *M*_s_ of samples was shown to change, as well as the proportion of oxygen vacancies.

## Competing interests

The authors declare that they have no competing interests.

## Authors’ contributions

SP designed and carried out all the experiments and data analysis, and participated in preparing the draft of the manuscript. SP co-supervised the research and offered technical support for TEM. PC and YP offered technical support for XANES measurement and analysis. SM, the project coordinator, supervised the research, designed the experiments, participated in preparing the draft of the manuscript, and revised the manuscript. All authors read and approved the final manuscript.
